# Molecular mechanisms driving *Aspergillus fumigatus* disease: host interaction, virulence, biofilms, and resistance

**DOI:** 10.3389/fmolb.2026.1829493

**Published:** 2026-06-23

**Authors:** Afsona Parveen, Prithviraj Karak, Atin Adhikari, Sayandeep K. Das

**Affiliations:** 1 Department of Bachelor in Medical Laboratory Technology, Durgapur Institute of Paramedical Science, Durgapur, West Bengal, India; 2 Department of Physiology, Bankura Christian College, Bankura, India; 3 Department of Biostatistics, Epidemiology, and Environmental Health Sciences, Jiann-Ping Hsu College of Public Health, Georgia Southern University, Statesboro, GA, United States; 4 Department of Pathology, Shri B. M. Patil Medical College Hospital and Research Centre, BLDE (Deemed to be University), Vijayapura, Karnataka, India

**Keywords:** antifungal resistance, aspergillosis, *Aspergillus fumigatus*, biofilm, diagnostics, pulmonary aspergillosis

## Abstract

**Introduction:**

*Aspergillus fumigatus* is a prevalent fungus that poses significant health dangers, especially for people with weakened immune systems. It may result in multiple conditions, such as allergic reactions, chronic pulmonary aspergillosis, and invasive aspergillosis, mainly impacting individuals with chronic lung diseases.

**Aims and objectives:**

The main goals of this review article are to grasp the recent advancements in the knowledge of cellular and molecular mechanisms related to *A. fumigatus* pathogenesis, create enhanced diagnostic methods, and assess current therapies.

**Methods:**

This review emphasizes the importance of biofilms in maintaining infections and enhancing antifungal resistance. Human infections due to *A. fumigatus* offer chances for further investigation, and these developing research topics are examined. Progress in diagnosis and treatment includes the creation of novel antifungal medications, customized medical strategies, and innovative diagnostic technologies. The relationships between the host and fungi, as well as the pathophysiology, must be understood.

**Results:**

The virulence factors produced during *A. fumigatus* pathogenesis can harm respiratory epithelial cells and impair immune function. It can survive and grow at temperatures up to 50 °C and release toxic materials that can harm human tissues and weaken the immune system. Public health initiatives and environmental stewardship are examples of preventive strategies. The development of vaccines can safeguard groups at high risk.

**Conclusion:**

Research on *A. fumigatus* pathogenesis reveals complex interactions between fungus and host cells. *Aspergillus fumigatus* is a global concern, with resistance rates increasing in Asia. New antifungal medicines, lipopeptides, and marine bioactive substances are being investigated to combat infections and improve health.

## Graphical Abstract

**Figure:**
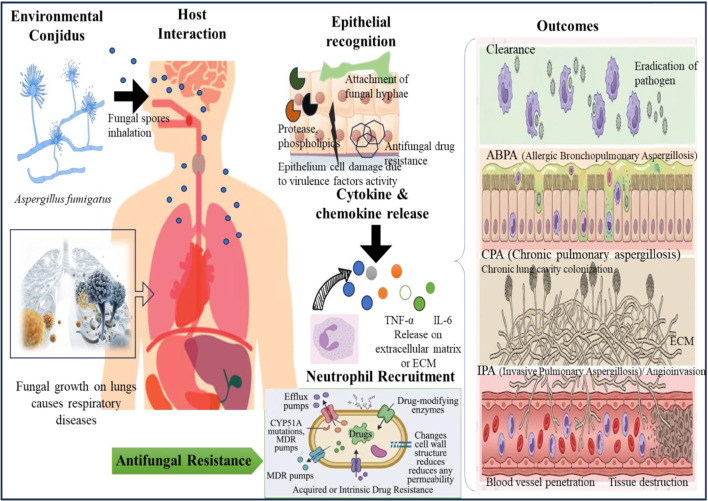

## Highlights

• Health effects of *A. fumigatus* include allergies to invasive aspergillosis.

• New biomarkers and diagnostic tools are urgently needed to diagnose infections.

• Rising antifungal resistance in *A. fumigatus* requires advanced combat strategies.

• Biology, host-pathogen interactions, and resistance mechanisms require attention.

• Some promising diagnostic tools and unexplored antifungal targets are reviewed and discussed.

## 1 Introduction

*Aspergillus fumigatus* is an environmentally ubiquitous, thermotolerant saprophytic mold and remains the principal etiologic agent of human aspergillosis, a disease spectrum that ranges from airway colonization and hypersensitivity syndromes to chronic pulmonary infection and rapidly progressive invasive disease (Latgé, 1999; Dagenais and Keller, 2009). The organism thrives in decaying organic matter and disperses abundant airborne conidia that are routinely inhaled; in most hosts these exposures are cleared without consequence, but disease emerges when local airway defenses are disrupted, when lung architecture is abnormal, or when systemic immune competence is impaired (Dagenais and Keller, 2009). Although classical patient groups include those receiving intensive chemotherapy, transplant recipients, and individuals with advanced HIV/AIDS, an expanding set of clinical contexts with chronic lung disease and iatrogenic immunomodulation has increased the relevance of *A. fumigatus* across routine respiratory medicine (Denham et al., 2019; Latgé and Chamilos, 2019).

After inhalation, conidia deposit along the conducting airways and alveoli, where early outcomes are shaped by mucociliary clearance, epithelial barrier function, and innate immune recognition (Cramer et al., 2011). Conidial swelling and germination initiate hyphal growth, and the ensuing host–fungus interface reflects a dynamic balance between antifungal effector responses and fungal strategies that limit killing, dampen inflammation, and promote tissue penetration (Cramer et al., 2011; Heinekamp et al., 2014). When containment fails, hyphae can invade the respiratory epithelium and parenchyma, driving inflammation and tissue injury and, in severe forms, enabling vascular invasion and dissemination, while chronic cavitary disease can develop in structurally abnormal lungs even in the absence of profound systemic immunosuppression (Dagenais and Keller, 2009; Deutsch et al., 2019).

**FIGURE 1:**
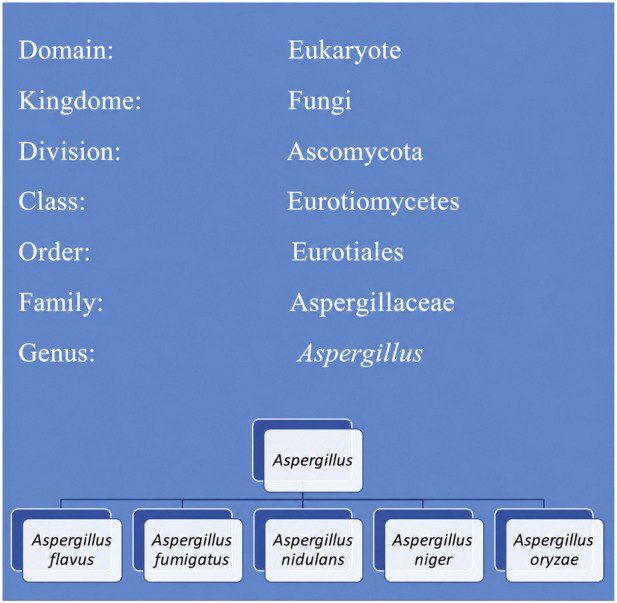
Scientific classification of *Aspergillus* fungi.

Pathogenic success is supported by a coordinated virulence repertoire that includes secondary metabolites, surface and cell-wall associated features, and secreted enzymes that collectively facilitate adhesion, nutrient acquisition, immune interference, and tissue damage (Hohl and Feldmesser, 2007; Sugui et al., 2007). These phenotypes are not constitutive; they are modulated by environmental and host-imposed stresses through conserved signaling and transcriptional programs. Temperature, pH, nutrient availability, oxidative stress, and hypoxia influence virulence expression, and regulatory networks such as cAMP–PKA signaling, G-protein coupled receptor pathways, and transcription factor–driven gene programs integrate these cues to govern growth, stress tolerance, secondary metabolite production, and pathogenicity (Liebmann et al., 2004; Bultman et al., 2016; Filho et al., 2020; Earle et al., 2023). This regulatory architecture provides a mechanistic basis for the heterogeneity of clinical outcomes observed across different host settings and lung microenvironments (Figure 1).

A further determinant of persistence and treatment failure is biofilm growth, in which multicellular hyphal networks are embedded within a protective extracellular matrix that promotes adherence, shields fungal elements from host defenses, and reduces antifungal efficacy (Morelli et al., 2021) Biofilm-associated states are clinically relevant in chronic airway and cavitary disease and in selected device-associated settings, where structural protection and altered physiology contribute to chronicity and relapse despite therapy (Jabra-Rizk et al., 2004; Rather et al., 2021; Mishra et al., 2023; Sharma et al., 2023). In parallel, azole resistance has emerged as a major clinical threat, driven by both patient-level selections during prolonged therapy and environmentally selected resistance linked to fungicide exposure, with surveillance limitations and phenotypic-only detection contributing to underestimation of the problem (Kang et al., 2025).

This review synthesizes contemporary mechanistic insights into early airway host–pathogen interactions, fungal immune evasion and virulence regulation, biofilm-associated persistence, and antifungal resistance, and integrates these mechanisms with clinicopathological patterns and current diagnostic and therapeutic strategies. Particular emphasis is placed on how molecular and cellular pathways explain transitions across the pulmonary aspergillosis spectrum and on the implications for next-generation diagnostics and resistance-aware management (Kang et al., 2025).

## 2 Methodology

This narrative review was prepared through a structured literature search of PubMed/MEDLINE, Scopus, Web of Science, and Google Scholar. The search covered literature from database inception to May 2026, with particular emphasis on recent diagnostic, resistance, and therapeutic literature published within the last 10 years. Search terms included combinations of “*A. fumigatus*,” “pulmonary aspergillosis,” “invasive aspergillosis,” “chronic pulmonary aspergillosis,” “allergic bronchopulmonary aspergillosis,” “ABPA,” “host–pathogen interaction,” “virulence,” “gliotoxin,” “MAPK,” “biofilm,” “azole resistance,” “*cyp51A*,” “galactomannan,” “β-D-glucan,” “PCR,” “molecular diagnostics,” and “antifungal therapy.”

Peer-reviewed original research articles, clinical guidelines, systematic reviews, diagnostic-accuracy studies, resistance-surveillance studies, and mechanistic studies relevant to *A. fumigatus* pathogenesis, host interaction, biofilm biology, antifungal resistance, diagnosis, and treatment were prioritized. Non-*fumigatus* studies were included only when they provided directly relevant mechanistic, diagnostic, or therapeutic context that could be applied cautiously to *A. fumigatus*. Reference lists of key guidelines and review articles were screened manually for additional relevant studies.

Because this article is a narrative review rather than a systematic review or meta-analysis, formal risk-of-bias assessment, protocol registration, and pooled quantitative synthesis were not performed. The findings should therefore be interpreted as a critical synthesis of selected mechanistic, clinical, diagnostic, and therapeutic literature rather than as an exhaustive systematic evaluation of all available evidence.

## 3 Early host–pathogen interaction at the airway interface

### 3.1 Airway epithelial sensing during the first 6–24 h of infection

Inhaled *A. fumigatus* conidia initially interact with the airway epithelium, where mucociliary clearance, epithelial barrier integrity, and early inflammatory signaling determine whether exposure is eliminated, establishes transient colonization, or progresses toward tissue invasion. A central mechanistic principle is that fungal ligand visibility is dynamic; as conidia swell and initiate germination, cell-wall remodeling increases exposure of immunostimulatory determinants such as β-glucans, thereby amplifying epithelial and myeloid sensing and shaping the kinetics of downstream recruitment (Croft et al., 2016).

Airway epithelial cells participate in fungal recognition through multiple pattern-recognition systems, including TLR-linked pathways and C-type lectin-associated signaling modules, and translate these inputs into chemokine and cytokine output that is functionally coupled to neutrophil recruitment. In this setting, epithelial-derived CXCL8/IL-8–centered programs and related chemokine networks act as early determinants of inflammatory cell influx and therefore influence whether fungal growth is contained at the luminal surface or gains an opportunity to expand into tissue compartments (Croft et al., 2016).

The epithelial response is also clinically consequential because it can bias immune polarization and tissue pathology. Integrated analyses of cytokine and chemokine circuits in aspergillosis emphasize that early innate signaling calibrates both amplification and immunopathology, providing a mechanistic bridge between early recognition events and later clinical phenotypes that range from hypersensitivity-dominant disease to invasive infection in susceptible hosts (Shankar et al., 2024).

Barrier integrity is a further outcome modifier at this stage. Experimental studies show that *A. fumigatus* secreted factors, including allergen and protease activity, can disrupt bronchial epithelial barrier properties, supporting the concept that epithelial damage can be an enabling step for disease escalation rather than a late epiphenomenon (Dunne et al., 2023). Conversely, epithelial cells can exert antifungal activity that limits germination, indicating that the epithelial layer can function as an active constraint on the conidia-to-hypha transition that is required for tissue invasion (Richard et al., 2018).

These early epithelial programs provide a mechanistic explanation for divergent outcomes. In airway ecosystems predisposed to mucus hypersecretion and type 2 inflammations, epithelial injury and danger signaling can amplify mucus plugging and inflammatory dysregulation, favoring hypersensitivity-predominant presentations. When epithelial injury coexists with impaired innate effector function, failure to suppress germination permits hyphal expansion and increases the probability of invasive trajectories (Philippe et al., 2003).

### 3.2 Innate effector modules that determine containment or progression

Alveolar macrophages constitute a principal early defense by phagocytosing conidia and suppressing germination. Mechanistic work demonstrates that conidial killing is strongly linked to reactive oxidant intermediate generation after conidial swelling, and that corticosteroid exposure can impair oxidant-dependent killing, providing a direct functional basis for steroid-associated susceptibility beyond epidemiologic association (Philippe et al., 2003).

Neutrophils become increasingly important once germination progresses, because hyphae are not readily eliminated by classical phagocytosis. Human immunologic analyses support the concept that neutrophils deploy distinct antifungal mechanisms against conidia *versus* hyphae, reinforcing a stage-specific logic in which the timing of neutrophil failure critically influences phenotype, including the transition from contained exposure to invasive disease (Gazendam et al., 2016).

Oxidative burst competence is a recurring axis across macrophage and neutrophil antifungal function, and mechanistic syntheses of immune interference by *A. fumigatus* emphasize that fungal survival strategies frequently converge on undermining phagocyte oxidative and non-oxidative killing pathways (Heinekamp et al., 2014).

Neutrophil extracellular traps (NETs) represent an additional effector program. *In vivo* experimental evidence indicates that NETs contribute substantially to containment and limitation of fungal spread rather than serving as a dominant fungicidal mechanism, which helps explain why NET formation alone does not reliably prevent invasive disease in profoundly immunocompromised hosts (Bruns et al., 2010).

Taken together, corticosteroid-mediated impairment of macrophage oxidant-dependent conidial killing and neutropenia-driven loss of hyphal-directed effector capacity define a mechanistic gateway by which epithelial exposure and colonization can convert into angioinvasive growth in susceptible host contexts (Philippe et al., 2003).

### 3.3 Immune evasion and immunomodulatory metabolites at the airway interface

**FIGURE 2:**
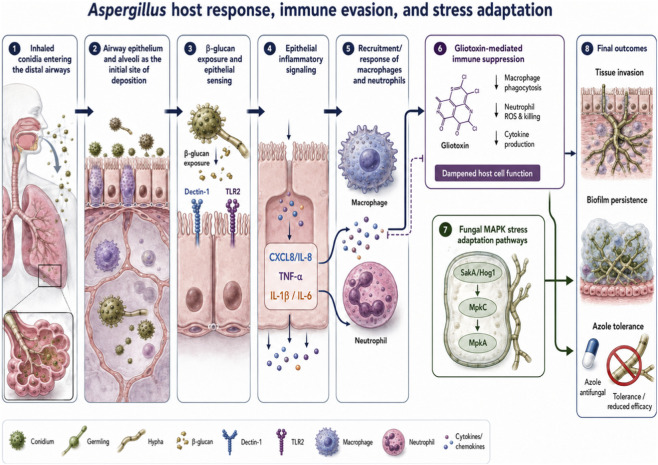
Pulmonary pathogenesis of *Aspergillus fumigatus* infection. Inhaled conidia interact with airway epithelial cells and innate immune cells, triggering cytokine/chemokine signaling and neutrophil recruitment. Fungal virulence factors such as gliotoxin impair phagocyte function, while MAPK-linked stress-response pathways support survival, gliotoxin regulation, and adaptation to host-imposed oxidative, osmotic, and cell-wall stress. Disease outcomes include clearance, allergic airway disease, chronic biofilm-associated persistence, or invasive pulmonary aspergillosis depending on host immunity and lung architecture.

*Aspergillus fumigatus* deploys immunomodulatory metabolites and surface features that reshape phagocyte signaling, intracellular processing, and epithelial integrity, thereby shifting the host–pathogen balance toward persistence or invasion. This interactional framing is useful because it separates mechanisms that sustain colonization in structurally abnormal niches from those that enable deeper tissue penetration and vascular invasion in high-risk hosts (Figure 2) (Heinekamp et al., 2014).

At the airway surface, protease and allergen activity can amplify epithelial injury and pro-inflammatory loops, which is consistent with clinical phenotypes characterized by mucus plugging and exaggerated inflammation when host background supports hypersensitivity (Osherov, 2012). The same class of epithelial-disruptive mechanisms can also lower local containment thresholds, particularly when combined with impaired innate effector function (Dunne et al., 2023).

In invasion-enabling contexts, gliotoxin provides a mechanistically defined example of direct immune suppression. Experimental work demonstrates that gliotoxin can impair macrophage phagocytic function through disruption of phosphoinositide signaling required for effective engulfment and integrin-linked cytoskeletal programs, thereby weakening early containment and facilitating progression (Schlam et al., 2016).

Conidial melanin represents an additional immune evasion strategy. A mechanistic study showed that *Aspergillus* melanin can inhibit LC3-associated phagocytosis by preventing recruitment of an NADPH oxidase subunit to the phagosome, linking a defined surface feature to suppression of a key antifungal pathway and increased virulence (Akoumianaki et al., 2016).

As hyphae develop, tissue invasion requires more than immune evasion; it also involves physical traversal of epithelial barriers. Experimental evidence supporting epithelial barrier crossing through host cytoskeletal remodeling provides a direct mechanistic bridge from airway surface interaction to deeper tissue involvement (Fernandes et al., 2018).

### 3.4 Virulence regulation networks linking environmental cues to pathogenic phenotypes

Virulence-associated phenotypes in *A. fumigatus* are governed by integrated signaling and transcriptional programs that convert environmental and host-imposed stresses into coordinated outputs relevant to disease, including growth kinetics, stress tolerance, secondary metabolite production, and host interaction traits. This regulatory framing is essential for interpreting why clinical outcomes vary across different lung microenvironments and immune states (Bultman et al., 2016).

The cAMP–PKA network is a core regulator of development and virulence, and genetic perturbation of this pathway alters conidiation and attenuates virulence, consistent with a model in which cAMP–PKA supports pathogenic fitness during immune confrontation (Liebmann et al., 2004).

Stress-activated MAPK modules further coordinate adaptation to oxidative, osmotic, and cell-wall stresses encountered *in vivo*. The SakA/Hog1 pathway and its associated MAPK components contribute to stress tolerance and virulence-linked fitness, and nuclear translocation behavior under stress underscores their role in rapid adaptive responses during host confrontation (Bruder Nascimento et al., 2016). The cell wall integrity MAPK MpkA is similarly implicated in oxidative stress response and virulence-relevant outputs, including regulation of secondary metabolite programs (Jain et al., 2011). Mechanistic linkage between stress signaling and immunomodulatory metabolite output is further supported by work connecting MAPK signaling with gliotoxin production and self-protection (Alves de Castro et al., 2024).

Within invasive lesions, hypoxia is a dominant selective pressure. SrbA, and SREBP-family transcription factor, is required for hypoxia adaptation and is tightly linked to azole susceptibility and virulence, positioning hypoxia-response circuitry as a mechanistic nexus that connects environmental adaptation with treatment-relevant phenotypes (Willger et al., 2008).

Iron limitation represents another host-imposed constraint, and HapX-mediated adaptation to iron starvation is crucial for virulence, coordinating repression of iron-consuming programs while promoting acquisition strategies such as siderophore-mediated uptake (Schrettl et al., 2010).

Transcription factor networks bridge development, adhesion, and host interaction traits. Developmental regulators control conidiation-linked gene programs that intersect with host-facing phenotypes (Dhingra et al., 2012). Adhesion and colonization traits relevant to airway persistence and biofilm initiation are regulated by factors such as MedA and SomA, linking transcriptional control to host interaction and virulence (Gravelat et al., 2009). Secondary metabolite regulation is coordinated in part by the Velvet complex component VeA, which influences conidiation and gliotoxin production, thereby connecting environmental and developmental regulation to immunomodulatory output.

## 4 Risk factors for *Aspergillus fumigatus* infection

### 4.1 Host and immune risk factors

The risk of *A. fumigatus* disease is determined by the interaction between inhaled environmental conidia, airway clearance mechanisms, lung architecture, and systemic immune competence. Because *A. fumigatus* produces abundant airborne conidia, exposure is common; however, disease develops only when host defenses fail to clear inhaled spores or when local pulmonary conditions permit colonization, germination, or invasion (O’Gorman, 2011). In immunocompetent individuals, mucociliary clearance, alveolar macrophages, and neutrophils usually prevent progression beyond transient exposure.

Impaired innate immunity is therefore one of the most important risk determinants. Alveolar macrophages provide early defense by phagocytosing conidia and limiting germination, while neutrophils become essential once hyphal growth begins. Neutropenia, particularly when severe or prolonged, markedly increases susceptibility to invasive pulmonary aspergillosis because hyphae cannot be effectively contained. Defects in phagocyte function, corticosteroid exposure, chemotherapy-related immunosuppression, hematologic malignancy, and advanced HIV/AIDS are therefore clinically important host-related risk factors (Kousha et al., 2011; Ewald et al., 2021).

### 4.2 Transplantation and iatrogenic immunosuppression

Solid-organ transplantation and other forms of iatrogenic immunosuppression increase the risk of invasive aspergillosis by reducing conidial clearance and impairing antifungal effector responses. In liver-transplant recipients, this risk is particularly relevant because postoperative immunosuppression, prolonged hospitalization, intensive-care exposure, broad-spectrum antimicrobial use, and impaired host defenses can converge during the early post-transplant period. A case-control study involving 260 individuals showed that liver-transplant recipients are more likely to develop *A. fumigatus* infection (Fortún et al., 2002).

Exposure in liver-transplant recipients is usually not attributable to a single unusual event. Rather, patients inhale airborne *A. fumigatus* conidia from ubiquitous environmental reservoirs such as dust, soil, decaying organic matter, construction activity, and hospital air systems. In most individuals, these exposures are inconsequential; however, transplant-related immunosuppression and postoperative vulnerability reduce the ability to eliminate inhaled conidia, allowing colonization, germination, or tissue invasion after otherwise common environmental exposure.

### 4.3 Environmental and occupational factors

Environmental exposure is central to *A. fumigatus* disease because the organism is widely present in soil, compost, decaying vegetation, organic debris, indoor dust, and air. Its small airborne conidia can remain suspended and reach the distal airways after inhalation. Exposure may increase during seasonal variation, agricultural activity, handling of plant material, composting, construction, renovation, and disturbance of contaminated dust. In healthcare environments, building work, inadequate containment, poor air handling, and insufficient protection of high-risk units can increase the risk of exposure among immunocompromised patients (Sabino et al., 2019; Raposo Puglia et al., 2024).

Occupational exposure is also relevant in individuals who work with organic dust, soil, plant material, waste, compost, or environments with high fungal spore burden. These exposures do not necessarily cause disease in healthy hosts, but they may become clinically important in individuals with chronic lung disease, impaired immunity, or repeated high-level exposure. In addition, environmental use of azole fungicides can contribute to selection pressure for resistant *A. fumigatus* strains, creating an ecological link between environmental exposure and clinically relevant antifungal resistance (Raposo Puglia et al., 2024).

### 4.4 Chronic lung disease and systemic comorbidity

Several pre-existing conditions are associated with increased susceptibility to *A. fumigatus* colonization or disease, including chronic obstructive pulmonary disease, asthma, cystic fibrosis, bronchiectasis, prior tuberculosis-related cavities, liver failure, decompensated cirrhosis, sepsis, and severe viral pneumonia (Kluge et al., 2021). These conditions increase risk through different mechanisms. Structural lung disease provides abnormal airway or cavitary niches where conidia can persist, germinate, and form chronic fungal burdens, while systemic illness and critical care can impair immune function and airway clearance.

The clinical consequence of these comorbidities varies across the aspergillosis spectrum. In asthma and cystic fibrosis, repeated antigen exposure can support allergic bronchopulmonary aspergillosis. In COPD, severe viral pneumonia, sepsis, or liver failure, impaired host defense and critical illness may increase the risk of invasive or subacute disease. In patients with structural lung damage, especially cavities or bronchiectasis, persistent colonization may progress toward chronic pulmonary aspergillosis or aspergilloma. Studies using serum galactomannan and clinical-risk assessment have also emphasized that pre-existing pulmonary and systemic conditions can increase the likelihood of clinically significant aspergillosis when compatible symptoms and imaging findings are present (Guinea et al., 2010).

### 4.5 Resistance-related risk and surveillance limitations

Antifungal resistance is an additional risk modifier because resistant *A. fumigatus* infection can compromise standard azole-based treatment. Resistance may emerge during prolonged antifungal exposure in patients with chronic or recurrent disease, or it may be acquired from the environment through inhalation of resistant conidia. The environmental route is particularly important because agricultural and environmental azole exposure can select resistant strains outside the patient, allowing resistant infection even in individuals without prior medical azole therapy.

The true burden of resistant *A. fumigatus* remains difficult to define because surveillance practices vary widely across regions and many centers still depend on phenotypic culture-based methods. Culture may be negative despite disease, susceptibility testing may not be routinely performed, and molecular resistance testing may not be available in all settings. As a result, resistance prevalence may be underestimated, especially in chronic respiratory disease populations and in regions where routine environmental and clinical surveillance is limited. Therefore, resistance-related risk should be interpreted through a combined clinical, microbiologic, and epidemiologic framework rather than as a purely laboratory finding (Zubovskaia, 2025).

## 5 Clinicopathological spectrum of pulmonary *Aspergillus fumigatus* disease

The clinicopathological spectrum of pulmonary aspergillosis is best interpreted by integrating three axes: (1) host immune competence, (2) pre-existing lung architecture (for example, cavities or bronchiectasis), and (3) depth of fungal invasion (airway luminal growth *versus* parenchymal and vascular invasion). This integrated approach mirrors consensus diagnostic frameworks that combine host factors, clinicoradiologic patterns, and mycologic evidence, and it clarifies why similar-appearing hyphae in tissue can represent either colonization or a life-threatening invasive infection.

### 5.1 Mechanistic bridge (host–fungus programs across the spectrum)

Across the spectrum, clinical phenotype reflects how early airway sensing and innate effector function intersect with fungal fitness programs. In ABPA, airway colonization and antigen exposure in a susceptible host preferentially amplifies type 2 inflammation (Th2-skewing, eosinophilia, mucus hypersecretion) driven by fungal allergens and proteases rather than tissue invasion. In CPA/aspergilloma, disease persistence is promoted by abnormal lung niches (cavities, pleural thickening, bronchiectasis) where fungal growth can organize into biofilm-like communities and chronic inflammation, with limited or absent invasion until transition states occur. In SAIA (chronic necrotizing/subacute invasive disease), intermediate immune impairment permits true tissue invasion over weeks to months with necrotizing inflammation. In acute invasive pulmonary aspergillosis (IPA), failure of macrophage and neutrophil containment enables angioinvasion, thrombosis, hemorrhagic infarction, and rapid dissemination (McClenny, 2005).

### 5.2 ABPA (hypersensitivity-predominant disease; usually noninvasive)

In ABPA, histopathology most often reflects allergic airway injury rather than parenchymal invasion. Specimens from mucus plugs or bronchial material typically show allergic mucin with abundant eosinophils and Charcot–Leyden crystals; fungal hyphae may be present within mucus plugs, often without invasion of viable tissue. Because ABPA is defined by a clinico-immunologic construct, pathology should be interpreted alongside fungal sensitization testing, total IgE, eosinophil counts, and imaging findings, consistent with the revised ISHAM guidance.

### 5.3 Chronic pulmonary aspergillosis (CPA), including aspergilloma, CCPA, CFPA, and aspergillus nodule

CPA typically arises in structurally abnormal lungs and is defined by chronicity (generally ≥3 months), compatible thoracic imaging (cavities with or without fungal ball, pleural thickening, fibrosis, or nodules), and microbiologic, serologic, or histologic evidence of Aspergillus involvement. Pathology varies by subtype: in simple aspergilloma, fungal elements are usually confined to cavity contents or necrotic debris, while the cavity wall shows chronic inflammation and fibrosis; the key reporting pivot is whether there is invasion of viable tissue. In CCPA/CFPA, progressive cavitation and fibrosis dominate, and histology should document the degree of chronic inflammation, fibrosis, and any transition toward invasive growth. Aspergillus nodules are an important surgical pathology mimics of malignancy or granulomatous infection; histology often demonstrates a necrotic nodule containing fungal hyphae, and again the invasion status should be explicitly stated (Schweer et al., 2014).

### 5.4 SAIA (subacute invasive)/chronic necrotizing pulmonary aspergillosis (CNPA)

SAIA represents a clinically crucial “border zone” in which disease progresses over weeks to a few months and demonstrates tissue invasion in a host with mild to moderate immunodeficiency or significant chronic lung disease. Histologically, SAIA/CNPA is characterized by necrotizing inflammation with hyphae invading lung parenchyma, often best highlighted with fungal stains (GMS/PAS), and it should be distinguished from noninvasive cavity colonization because it typically warrants more aggressive antifungal management than chronic colonizing forms (Denning et al., 2016).

### 5.5 Acute invasive pulmonary aspergillosis (IPA), including angioinvasive disease

IPA is the most severe pulmonary presentation and is most strongly associated with profound immune compromise (for example, neutropenia, hematologic malignancy, transplantation, intensive immunosuppression). The defining pathology feature is invasion of viable tissue, and the highest-risk pattern is angioinvasion, where hyphae invade vessel walls and lumina, producing thrombosis, hemorrhage, and infarction with potential hematogenous dissemination. In suspected IPA, pathology should be interpreted within the EORTC/MSG consensus logic (host factor + clinical features + mycologic evidence) used to categorize disease certainty (proven/probable/possible) in research and practice, and reports should communicate urgency given guideline emphasis on early effective antifungal therapy (Table 1).

**TABLE 1:** Clinico–patho–mechanism map of pulmonary aspergillosis.

Clinical entity	Usual host setting	Clinicoradiologic pattern	Pathology hallmark (what to look for)	Dominant host pathway	Fungal program (dominant)	Key diagnostic handles	Framework anchor
ABPA	Asthma, cystic fibrosis, bronchiectasis; fungal sensitization	Exacerbations, mucus plugging, bronchiectasis; serology-driven diagnosis	Allergic mucin, eosinophils, Charcot–Leyden crystals; hyphae in mucus plugs; usually no invasion	Type 2 inflammation (Th2), eosinophilia, mucus hypersecretion	Allergen/protease exposure; airway luminal growth	Total IgE, *A. fumigatus*–specific IgE/IgG, eosinophils; imaging	ISHAM ABPA guideline (Agarwal et al., 2024)
Aspergilloma (fungal ball)	Pre-existing cavity (post-TB, sarcoid, *etc.*)	Intracavitary mass ± hemoptysis	Fungal mass in cavity contents; invasion absent or minimal	Structural niche with local immune constraints	Biofilm-like growth in cavity; chronic persistence	Imaging + microbiology/serology; histology to assess invasion	ERS/ESCMID CPA guidance (Denning et al., 2016)
CCPA/CFPA (CPA spectrum)	Structural lung disease; mild immunosuppression	Cavities ± pleural thickening, fibrosis; ≥3 months	Chronic inflammation/fibrosis; hyphae in cavity debris; assess invasion	Chronic inflammation with impaired clearance	Persistence, stress adaptation; possible biofilm matrix	Aspergillus IgG, culture/PCR; histology when available	ERS/ESCMID CPA guidance (Agarwal et al., 2024)
SAIA/CNPA	Mild–moderate immunodeficiency; chronic lung disease	Progressive cavitary/consolidative disease over weeks–months	Tissue invasion with necrotizing inflammation; hyphae in necrotic lung	Partial failure of innate containment	Invasive growth with stress-response fitness	Histology (invasion), culture/PCR; biomarkers supportive	CPA/SAIA positioning in ERS/ESCMID and reviews (Agarwal et al., 2024)
Acute IPA (angioinvasive)	Profound immunosuppression (neutropenia, HSCT/SOT, *etc.*)	Nodules, halo sign; rapid progression in high-risk host	Angioinvasion, thrombosis, hemorrhagic infarction; necrosis	Innate effector failure (neutrophils/macrophages)	Angioinvasive tissue penetration, dissemination	GM/BDG, PCR, culture; histology for “proven”	EORTC/MSG definitions; IDSA therapy guidance (Agarwal et al., 2024)
Aspergillus nodule	Often non-neutropenic; incidental nodule	Solitary/multiple nodules mimicking tumor	Necrotic nodule with hyphae; invasion often absent	Localized inflammatory response	Local persistence in focal niche	Often requires histology for diagnosis	Aspergillus nodule review (Muldoon et al., 2016 )

Abbreviations: ABPA, allergic bronchopulmonary aspergillosis; CPA, chronic pulmonary aspergillosis; CCPA, chronic cavitary pulmonary aspergillosis; CFPA, chronic fibrosing pulmonary aspergillosis; SAIA, subacute invasive aspergillosis; CNPA, chronic necrotizing pulmonary aspergillosis; IPA, invasive pulmonary aspergillosis; HSCT, hematopoietic stem-cell transplantation; SOT, solid-organ transplantation; GM, galactomannan; BDG, β-D-glucan; PCR, polymerase chain reaction.

#### 5.6 Practical pathology reporting anchors (to align tissue findings with molecular diagnostics)

In tissue sections, septate hyaline hyphae with acute-angle branching are strongly suggestive of Aspergillus but are not fully species-specific among hyaline septate molds; therefore, optimal reporting should describe (1) hyphal morphology, (2) presence or absence of tissue invasion, (3) vascular invasion, (4) necrosis and infarction, and (5) host response pattern, while explicitly recommending correlation with culture and/or molecular methods where available. This is particularly important because contemporary practice increasingly relies on non-culture diagnostics (for example, serum/BAL galactomannan, β-D-glucan, Aspergillus PCR) and resistance-marker detection, which complement histology and improve species-level inference and therapeutic targeting (Guarner and Brandt, 2011).

## 6 Clinical manifestations and diagnosis of *Aspergillus fumigatus* infection

Pulmonary disease attributable to *Aspergillus fumigatus* spans colonization, hypersensitivity-associated airway disease, chronic cavitary infection, and invasive aspergillosis, and the diagnostic problem is often not the detection of hyaline septate hyphae alone but the correct placement of the patient along this spectrum with respect to tissue invasion and host context (Schwartz and Thiel, 1997; Denning, 2001; Guinea et al., 2010; Schweer et al., 2014). Clinical presentations are frequently nonspecific. Chronic pulmonary aspergillosis typically evolves over months with constitutional symptoms, chronic cough, and dyspnea, while hemoptysis may occur and can be prominent in cavitary disease and aspergilloma (Denning, 2001; Schweer et al., 2014). In invasive disease, fever, pleuritic chest pain, hemoptysis, and progressive respiratory compromise can occur, and dissemination may involve the central nervous system and skin, particularly in profoundly immunocompromised hosts (Kuijer et al., 1992; Denning, 1998; Van Burik et al., 1998; Ozhak-Baysan et al., 2010). Because delayed recognition is associated with progression and higher mortality in invasive aspergillosis, diagnostic strategy should prioritize early, syndrome-directed sampling and rapid mycologic confirmation in patients with compatible clinical and imaging features (Denning, 1998; Van Burik et al., 1998).

Imaging is a critical gatekeeper for diagnostic probability and tissue targeting. Chest computed tomography provides actionable early information in patients with suspected invasive pulmonary aspergillosis and may demonstrate patterns that strengthen pre-test probability before microbiologic confirmation is available (Vandewoude and Vogelaers, 2007). The halo sign has been associated with early invasive disease in appropriate clinical settings and remains a useful radiologic clue when interpreted alongside mycologic testing rather than as an isolated determinant (Blum et al., 1994). Hybrid imaging strategies such as PET/CT with [18F]FDG can aid lesion localization and assessment of metabolic activity; however [18F]FDG is not specific for fungal infection and cannot reliably distinguish invasive fungal disease from malignancy or sterile inflammation, limiting its role as a discriminative diagnostic tool in routine practice (Thornton, 2018).

Definitive diagnosis often depends on the integration of microbiology with tissue-based pathology. Direct microscopy and culture remain foundational, but sensitivity varies by sample type and prior antifungal exposure, and culture positivity is not synonymous with invasive disease because colonization can occur, particularly in structurally abnormal lungs (McClenny, 2005). The contribution of the laboratory can be strengthened by optimizing specimen processing, improving morphologic recognition in stained preparations, and maximizing sporulation and growth conditions for *Aspergillus* species in culture (McClenny, 2005). Where feasible, bronchoalveolar lavage and tissue sampling guided by imaging provide higher-yield material for integrated cytology, histopathology, culture, and molecular testing, and also permit invasion assessment that cannot be inferred reliably from respiratory cultures alone. *Aspergillus fumigatus* can be cultured in the laboratory from appropriate respiratory or tissue specimens, although culture results must be interpreted with clinical and radiologic context because airway colonization can occur. Commonly used fungal media include Sabouraud dextrose agar, potato dextrose agar, malt extract agar, and Czapek-Dox agar, usually with antibacterial agents when respiratory specimens are processed. Incubation at both 25 °C–30 °C and 35 °C–37 °C can improve recovery and morphological assessment; growth is often rapid, and colonies typically become visible within a few days. Thermotolerance, including growth at human body temperature and survival/growth at higher temperatures, supports the pathogenic fitness of *A. fumigatus*. Microscopic identification should be correlated with culture morphology, lactophenol cotton blue preparation, and, where available, molecular confirmation or susceptibility testing. Traditional microscopy and culture remain essential even as molecular and immunologic tests expand diagnostic capacity (McClenny, 2005).

Biopsy and histopathology are particularly valuable when the clinical question is whether fungal elements represent colonization *versus* tissue-invasive disease, and when alternative etiologies such as malignancy, granulomatous disease, vasculitis, or organizing pneumonia are plausible differentials. Tissue examination also becomes pivotal in localized nodules or cavitary wall lesions where radiology may mimic tumor or tuberculosis-associated pathology, and where management depends on confirming fungal invasion or identifying a non-fungal primary process (Denning, 2001; Schweer et al., 2014). In suspected invasive aspergillosis, demonstration of tissue invasion and especially vascular invasion provides the highest level of diagnostic certainty and has immediate therapeutic implications (Guinea et al., 2010).

On histology, *Aspergillus* typically appears as hyaline, septate hyphae with acute-angle branching, but this morphology is not completely species-specific among hyaline molds, and a structured differential diagnosis should be maintained in every tissue-based report. Other hyaline septate molds such as *Fusarium* and *Scedosporium/Lomentospora* can produce similar-appearing hyphae, while mucormycosis is usually characterized by broader, ribbon-like, pauciseptate hyphae with irregular branching; nonetheless, morphologic overlap and tissue processing artifacts can mislead interpretation, particularly in necrotic samples. In addition, dematiaceous fungi may show pigmented hyphae, and *Candida* can show yeast forms and pseudohyphae, further widening the differential in necrotizing infections. For these reasons, pathologic reporting should explicitly document hyphal width, septation, branching pattern, pigment, angioinvasion, extent of necrosis, and the tissue compartment involved, and should avoid overconfident species-level assignment based on morphology alone when culture or molecular confirmation is not available. A practical approach is to report “hyaline septate mold consistent with aspergillosis” when morphology supports *Aspergillus* yet species confirmation is pending or unavailable, while emphasizing invasion status and urgency where appropriate (Guarner and Brandt, 2011).

Special stains are essential adjuncts for detection and characterization of fungi in tissue and cytology. Gomori methenamine silver (GMS) and periodic acid–Schiff (PAS) enhance visualization of fungal walls, increase detection in necrotic debris, and improve assessment of tissue and vascular invasion, especially when fungal burden is low. In heavily necrotic lesions, repeated deeper sections and targeted staining can be necessary to avoid false-negative interpretation, and the pathologist should correlate stain findings with the distribution of necrosis, inflammation, and hemorrhage to support the diagnosis of an angioinvasive mold infection (Guarner and Brandt, 2011).

Molecular diagnostics provide a complementary route to organism identification when culture is negative, slow, or compromised by antifungal exposure. PCR-based assays can detect *Aspergillus* DNA from clinical specimens and, when properly validated, can increase diagnostic yield and shorten time to actionable confirmation, particularly in respiratory samples (Barnes et al., 2018). In addition, the standardization of molecular tests that simultaneously detect *Aspergillus* and common azole-resistance markers has expanded diagnostic capability from “presence/absence” to early therapeutic stratification, which is increasingly relevant as resistance prevalence grows (Barnes et al., 2018). At the tissue level, fungal DNA sequencing approaches targeting conserved ribosomal loci (for example, ITS or 28S regions) can support species-level identification when formalin-fixed tissue is the only available specimen, although performance depends on DNA quality, fungal burden, and laboratory expertise; such strategies are particularly valuable when hyaline septate mold morphology is present but culture is not contributory (Barnes et al., 2018).

Non-culture biomarkers strengthen early diagnosis, particularly in invasive disease where therapeutic delay worsens outcomes. Galactomannan is a key biomarker for invasive aspergillosis and has been evaluated across serum and bronchoalveolar lavage contexts, with interpretation dependent on host background, sampling strategy, and competing causes of false positivity or negativity (Verdaguer et al., 2007). Comparative studies of β-D-glucan and galactomannan support their use as adjuncts rather than stand-alone tests, and emphasize the importance of integrating biomarker results with imaging and clinical probability (McClenny, 2005; Verdaguer et al., 2007; Soubani, 2024). The availability of commercial options and automated platforms has increased feasibility in many centers, but test selection should reflect local epidemiology, laboratory capacity, and patient population (Soubani, 2024).

In low-resource settings, a pragmatic diagnostic pathway should prioritize early clinical recognition, targeted imaging, and rapid microscopy while maintaining a low threshold for initiating confirmatory testing. High-yield approaches include careful assessment of risk context and symptom tempo, chest radiography as an initial screen with escalation to CT when feasible, and direct microscopy of respiratory specimens using fungal stains to rapidly detect hyphal elements. Where histopathology services exist, GMS/PAS staining of biopsy material can provide decisive evidence of invasion and guide urgent escalation of care, even when advanced molecular tests are not available. When biomarker assays or PCR are unavailable, structured reporting that clearly indicates the presence of an angioinvasive mold infection and recommends immediate clinical correlation and antifungal therapy can function as a critical trigger for time-sensitive management. The need for early diagnosis is particularly acute for invasive pulmonary aspergillosis, where progression can be rapid and delays in effective therapy are associated with poor outcomes, making “time-to-diagnosis” a determinant of survival rather than a purely academic metric (Schwartz and Thiel, 1997; Denning, 2001; Guinea et al., 2010).

Taken together, diagnosis of *A. fumigatus* infection should be approached as a clinicopathological integration problem in which imaging localizes disease and stratifies probability (Blum et al., 1994; Vandewoude and Vogelaers, 2007; Thornton, 2018), microbiology and biomarker testing provide organism-level evidence (McClenny, 2005), and histopathology adjudicates invasion and refines the differential diagnosis when morphology overlaps among molds. This framework sets the basis for the subsequent treatment section, where antifungal selection, the urgency of initiation, and the consideration of surgical or adjunctive strategies depend directly on syndrome classification, invasion status, and the strength of mycologic evidence.

## 7 Treatment and management of *Aspergillus fumigatus* infections

Therapeutic strategy for *A. fumigatus* disease must be aligned with syndrome classification because antifungal priorities, duration of therapy, and the role of adjunctive interventions differ substantially across allergic, chronic, and invasive presentations. In invasive aspergillosis, early initiation of effective systemic antifungal therapy remains a central determinant of outcome, and delay in appropriate therapy is associated with progression and excess mortality (Patterson et al., 2016). Treatment selection is interpreted alongside the diagnostic category, as a positive microbiologic result may indicate colonization, allergic disease, chronic infection, or invasive disease depending on host context and radiologic findings (Table 2).

**TABLE 2:** Current diagnostic approaches for *Aspergillus fumigatus* disease and their treatment relevance.

Specimen/Starting material	Diagnostic tool	Best clinical use	Efficacy/Limitation to state	Treatment relevance/Commonly linked drugs	Key reference
Serum	Galactomannan antigen assay	Screening and diagnostic support for invasive aspergillosis, especially in hematologic malignancy and HSCT settings	Serum GM has moderate diagnostic accuracy; pooled sensitivity and specificity were reported as 0.71 and 0.89, respectively, in a meta-analysis. Performance is lower in some non-neutropenic and solid-organ transplant populations and can be affected by mold-active prophylaxis	Supports early mold-active therapy when interpreted with host risk and CT findings; commonly linked treatment options include voriconazole, isavuconazole, or liposomal amphotericin B depending on clinical context and resistance concern	Pfeiffer et al. (2006)
BAL fluid	BAL galactomannan antigen assay	Diagnosis of invasive pulmonary aspergillosis when respiratory sampling is feasible	BAL-GM has higher sensitivity than serum GM in many pulmonary cases. At a cutoff of 0.5, pooled sensitivity and specificity were 0.87 and 0.89, respectively; a cutoff of 1.0 may improve specificity	Supports early antifungal initiation in compatible host/radiology; commonly linked to voriconazole or isavuconazole first-line, with liposomal amphotericin B considered when azole resistance or intolerance is suspected	Zou et al. (2012)
Serum	1,3-β-D-glucan assay	Adjunctive marker for invasive fungal disease	Useful as an adjunct marker but not specific for Aspergillus; also positive in invasive candidiasis and several other fungal infections. Should be interpreted with imaging, host risk, and other mycologic tests	Should not independently determine drug choice; positive results may support escalation to mold-active therapy only when the clinical syndrome is compatible	Lamoth et al. (2021)
Sputum/BAL/bronchial wash/tissue	Direct microscopy and fungal culture	Detection of hyaline septate molds, species identification, and antifungal susceptibility testing	Culture is specific when positive but sensitivity is variable; respiratory culture may represent colonization rather than invasive disease. Culture remains essential for susceptibility testing	Positive culture plus compatible disease supports antifungal selection and susceptibility-guided therapy; azole-resistant isolates may require liposomal amphotericin B or specialist-directed alternatives	Barton (2013)
Lung biopsy/resection/cavitary material	Histopathology with H&E, GMS, and PAS stains	Confirmation of tissue invasion, angioinvasion, necrosis, and distinction between colonization and invasive disease	Histology can prove invasion but morphology alone is not species-specific; hyaline septate acute-angle branching moldsshould be correlated with culture, PCR, or sequencing where possible	Demonstration of tissue or vascular invasion supports urgent systemic mold-active therapy; localized aspergilloma or life-threatening hemoptysis may require surgical/source-control consideration	Sabino and Wiederhold (2022)
Chest CT	CT chest/HRCT	Early localization and pattern recognition in IPA, CPA, aspergilloma, and ABPA	CT is sensitive for lesion localization but not organism-specific. Halo sign, nodules, cavitation, air-crescent sign, bronchiectasis, mucus plugging, or fungal ball must be interpreted with host factors and mycology	Guides urgency of sampling and treatment; compatible CT in a high-risk host may justify early antifungal therapy while microbiologic confirmation is pursued	Greene et al. (2007)
BAL fluid	Aspergillus PCR	Rapid detection of Aspergillus DNA in suspected pulmonary aspergillosis	BAL PCR may improve early diagnosis. One large clinical study reported BAL PCR sensitivity and specificity of 88.6% and 95.5% for invasive aspergillosis; PCR detects fungal DNA but does not alone prove invasion	Helps early initiation or de-escalation when combined with GM, culture, and imaging; does not replace susceptibility testing	Imbert et al. (2018)
BAL fluid	AsperGenius/resistance-marker PCR	Detection of Aspergillus DNA plus common cyp51A-linked azole-resistance mutations	Detects common resistance markers such as TR34/L98H and TR46/Y121F/T289A directly from BAL. In a multicenter study, RAM detection was associated with azole treatment failure; limitation: panels may miss uncommon or non-cyp51A mechanisms	Supports early resistance-aware therapy. If resistance markers are detected, avoid uncritical azole monotherapy and consider liposomal amphotericin B or specialist-directed therapy	Chong et al. (2016)
Serum	Aspergillus-specific IgG	Diagnosis of chronic pulmonary aspergillosis and aspergilloma with compatible imaging and chronic symptoms	Useful in CPA; one study using histologically proven CPA as reference standard reported ImmunoCAPAspergillus-specific IgG sensitivity of 85.1% and specificity of 83.6%. False positives require radiologic correlation	Supports prolonged oral azole therapy in CPA, commonly itraconazole, voriconazole, or posaconazole depending on tolerance, interactions, and susceptibility	Salzer et al. (2023)
Serum ± CT	A. fumigatus-specific IgE, total IgE, eosinophil count, Aspergillus IgG, and CT features	Diagnosis and classification of ABPA	ABPA is an immunologic diagnosis; A. fumigatus-specific IgE is sensitive, while high-attenuation mucus on CT is highly specific. Culture alone is not adequate because ABPA reflects hypersensitivity rather than tissue invasion	Guides anti-inflammatory and antifungal strategy; commonly systemic corticosteroids and/or itraconazole depending on disease activity and guideline-based classification	Agarwal et al. (2013)
BAL fluid/blood	Metagenomic next-generation sequencing	Selected complex cases, culture-negative disease, mixed infection, or difficult host contexts	mNGS can add diagnostic information but requires careful interpretation because detection of DNA does not always prove invasive disease. BALF generally performs better than blood for pulmonary disease	May support early targeted antifungal therapy when conventional tests are negative or delayed; should be interpreted with GM, PCR, culture, imaging, and host risk	Xu et al. (2025)

For invasive aspergillosis, triazole therapy is the cornerstone of primary management. Voriconazole is widely supported as a first-line agent based on clinical efficacy and accumulated experience, while isavuconazole is an accepted alternative in settings where tolerability, drug–drug interaction profile, or specific safety considerations influence agent selection (Wingard and Leather, 2004; Patterson et al., 2016; Jenks and Hoenigl, 2018). Liposomal amphotericin B is an important alternative when azoles cannot be used, when there is concern for intolerance, or when local epidemiology raises suspicion for azole resistance (Wingard and Leather, 2004; Patterson et al., 2016). Posaconazole and echinocandins are generally positioned as salvage options or as components of stepwise management in refractory disease, with selection influenced by clinical trajectory, prior antifungal exposure, and resistance data when available (Patterson et al., 2016; Jenks and Hoenigl, 2018).

Echinocandins inhibit β-(1,3)-D-glucan synthase, thereby impairing fungal cell-wall synthesis, and are typically fungistatic against *Aspergillus*, which explains their common use as adjuncts rather than sole primary therapy (Sucher et al., 2009). Evidence for combination therapy in invasive aspergillosis is heterogeneous. Historical *in vitro* and clinical literature has reported potentially beneficial interactions for selected combinations, including amphotericin B with echinocandins such as micafungin, but overall strength of evidence remains moderate and context-dependent. Contemporary guideline-based approaches generally do not recommend routine combination therapy as universal first-line management; combination regimens are instead considered selectively in severe disease, in treatment failure, or when resistance or pharmacologic constraints limit effective monotherapy (Patterson et al., 2016).

Management of chronic pulmonary aspergillosis frequently requires prolonged therapy and careful longitudinal monitoring, and the feasibility of sustained treatment is often constrained by drug toxicity, tolerability, and clinically important drug–drug interactions (Russo et al., 2020; Takazono et al., 2025). Co-infection with non-tuberculous mycobacteria is increasingly recognized as a high-risk setting because overlapping long treatment courses, cumulative toxicity, and complex interaction profiles can compromise both antifungal and antimycobacterial regimens (Russo et al., 2020). In such patients, treatment planning should incorporate interaction checking, liver function monitoring, and a structured reassessment of response and tolerability at defined intervals (Russo et al., 2020; Takazono et al., 2025).

Surgical intervention is not the default treatment for invasive pulmonary aspergillosis, but it remains an important adjunct in carefully selected situations, including localized disease with life-threatening hemoptysis, resectable lesions causing ongoing sepsis despite antifungal therapy, and complications such as aspergilloma in suitable operative candidates (Denning and Stevens, 1990; Bongomin et al., 2021). Surgical procedures commonly include segmentectomy, lobectomy, or pneumonectomy depending on localization and pulmonary reserve, and risk is lower in patients without pleural or chest wall involvement (Csekeö et al., 1997; Bongomin et al., 2021). In extrapulmonary invasive disease, source control is often essential when anatomically feasible; valve replacement is a key component of management for *Aspergillus* endocarditis, and in ocular involvement, vitrectomy with intravitreal amphotericin B has been described as a necessary component of therapy in severe cases (Denning and Stevens, 1990).

Allergic bronchopulmonary aspergillosis is managed differently because pathology is driven primarily by immune dysregulation rather than tissue invasion. Systemic corticosteroids remain the mainstay of therapy to control inflammation, and oral azole therapy is used in many settings as an adjunct to reduce fungal burden and steroid requirements, with clinical monitoring commonly guided by symptoms, imaging, and trends in total serum IgE (Wang et al., 1979; Vlahakis and Aksamit, 2001; Hogan and Denning, 2011). Allergen avoidance is clinically important because repeated exposure to high spore burdens can precipitate exacerbations and perpetuate airway injury (Vlahakis and Aksamit, 2001).

Treatment-related complications are clinically significant and frequently limit duration or dosing intensity. Voriconazole is associated with reversible visual disturbances and hepatotoxicity, and longer courses can be complicated by photosensitivity and other cutaneous adverse effects; clinically relevant drug–drug interactions are common due to CYP-mediated metabolism, and dose optimization may require therapeutic drug monitoring in many settings (Abdel-Haq et al., 2014). Isavuconazole is generally regarded as having a comparatively favorable interaction profile among mold-active azoles, but it has a distinct cardiac electrophysiology signal, with clinical data demonstrating QT interval shortening rather than prolongation (Mellinghoff et al., 2018). Amphotericin B toxicity is dominated by nephrotoxicity and electrolyte wasting, and although liposomal formulations reduce nephrotoxicity compared with conventional amphotericin B deoxycholate, renal injury remains a clinically relevant risk that requires proactive monitoring and supportive measures (Karimzadeh et al., 2016). These adverse-effect profiles have direct management consequences in invasive disease, where antifungal choice, need for monitoring, and feasibility of prolonged therapy are dictated as much by host comorbidity and concomitant medications as by antifungal spectrum (Csekeö et al., 1997; Russo et al., 2020; Takazono et al., 2025).

This treatment framework follows directly from the diagnostic principles outlined in the preceding section: accurate syndrome assignment, early confirmation in suspected invasive disease, and resistance-aware testing where feasible are essential to selecting appropriate first-line therapy and avoiding preventable delays or ineffective regimens (Vlahakis and Aksamit, 2001).

## 8 Prevention and control of *Aspergillus fumigatus* infections

### 8.1 Healthcare engineering controls

Healthcare facilities can prevent and control *A*. *fumigatus* infections by implementing high-efficiency particulate air (HEPA) filters, frequent environmental cleaning, construction control, patient isolation, staff education, and surveillance (Vlahakis and Aksamit, 2001). HEPA filters minimize penetration of airborne *A. fumigatus*spores, and environmental cleaning disinfects surfaces, particularly in immunocompromised areas (Mareković, 2023).

### 8.2 Environmental monitoring and construction control

Environmental monitoring is essential for lowering exposure to *A. fumigatus*, particularly in high-risk environments such as hospitals, building sites, and among immunocompromised people. It entails detecting sources of exposure by air and surface samples, estimating risk using spore counts, and identifying species (Martony et al., 2019). Implementing control methods, such as HEPA filtration–as mentioned above, cleaning and disinfection, and utilizing proper building codes, can also help to reduce contamination (Ademe, 2020). Regular monitoring allows us to analyze the effectiveness of these strategies and identify areas for improvement (Ademe, 2020). Environmental monitoring can help high-risk settings limit *A. fumigatus* exposure and safeguard vulnerable people (Martony et al., 2019). Regular monitoring can assist in discovering areas for improvement and protect individuals’ safety in high-risk environments.

Construction-based control includes enclosing spaces and utilizing HEPA-filtered vacuums. Patient isolation places and rooms with immunocompromised patients should be equipped with HEPA filtration and positive air pressure (Mareković, 2023).

### 8.3 High-risk patient prevention and prophylaxis

Individuals at high risk, such as those with impaired immune systems, organ transplant recipients, and HIV/AIDS patients, can benefit from *A. fumigatus* infection prevention approaches (Hahn et al., 2002). These approaches include antifungal prophylaxis with posaconazole, voriconazole, or itraconazole, as well as echinocandins (Singh and Paterson, 2005). Environmental measures include avoiding high-level *A. fumigatus* spore exposure at building sites or sites with decomposing organic debris, as well as employing HEPA filters in residential areas (Vazquez, 2016). Immunomodulation with growth factors such as granulocyte-colony stimulating factor (G-CSF) or granulocyte-macrophage colony-stimulating factors (GM-CSF) can boost the immune system. Regular surveillance for symptoms of *A. fumigatus*infection is also required (Martony et al., 2019). High-risk persons can lower their risk of contracting A. fumigatus infections by following the above-mentioned preventive practices.

### 8.4 Clearance and disinfection of *Aspergillus fumigatus* reservoirs

Clearance of *A. fumigatus* in healthcare settings is primarily achieved by reducing airborne conidial exposure and removing environmental reservoirs rather than by patient “decolonization.” Practical measures include HEPA filtration for high-risk units, positive-pressure protected environments for selected immunocompromised patients, construction-risk assessment, sealed barriers during renovation, dust suppression, wet cleaning rather than dry sweeping, HEPA-filtered vacuuming, prompt removal of water-damaged or mold-contaminated materials, and routine cleaning of high-touch surfaces. Environmental and equipment surfaces should be cleaned and disinfected using compatible EPA-registered hospital disinfectants according to manufacturer instructions. Oxidizing agents such as sodium hypochlorite, hydrogen peroxide, chlorine dioxide, and peracetic acid are commonly discussed for fungal-spore inactivation, but surface compatibility, organic load, concentration, and contact time determine efficacy; therefore, institutional infection-control policy and product labeling should guide use. CDC environmental infection-control guidance emphasizes EPA-registered disinfectants, wet-dusting in immunocompromised patient areas, avoidance of dust-dispersing cleaning methods, and HEPA-filtered vacuums for areas with patients at risk (CDC, 2026; Martony et al., 2019; Ademe, 2020).

### 8.5 Vaccination and immunomodulation: investigational directions

Vaccination efforts against *A*. *fumigatus* are geared toward creating safe and effective vaccines for high-risk individuals. Vaccine options include live or heat-killed mutant conidia, dendritic cell-based vaccines, subunit vaccinations, and adjuvants. A study found that immunization with live or heat-killed *A. fumigatus*ΔsglA conidia effectively protected immunocompromised mice from invasive aspergillosis (Fernandes et al., 2022). Dendritic cell-based vaccines have shown potential in eliciting protective Th1 responses and boosting resistance to infection. Subunit vaccines use unique antigens to elicit protective immune responses. Adjuvants, such as CpG ODN, are being investigated to improve vaccine efficacy (Fernandes et al., 2022). Future directions include better understanding immune responses, creating vaccines for high-risk populations, and increasing vaccination efficacy. More research is required to create effective vaccinations for *A*. *fumigatus*.

## 9 *Aspergillus fumigatus* and drug resistance

**FIGURE 3:**
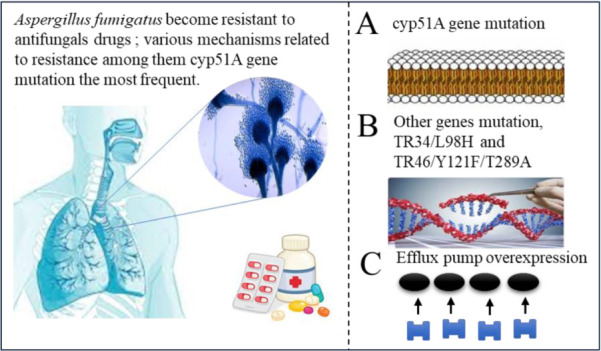
Various aspects of antifungal resistance in *Aspergillus fumigatus*. **(A)** cyp51A gene mutation associated with azole resistance. **(B)** Other resistance-associated mutations, including TR34/L98H and TR46/Y121F/T289A. **(C)** Efflux-pump overexpression leading to increased antifungal drug efflux and reduced intracellular drug concentration.

Numerous processes, such as target site mutations, efflux pump overexpression, biofilm development, genetic alterations, stress response, and hetero-resistance, can cause *A*. *fumigatus* to become resistant to antifungals (Parker et al., 2014; Fisher et al., 2022). Target site mutations in the cyp51A gene lower the binding affinity of azole antifungals, leading to azole resistance. Particular mutations, like TR34/L98H and TR46/Y121F/T289A, are frequently seen when target sites are mutated (Fisher et al., 2022) (Figure 3).

Overexpression of efflux pumps can increase efflux while decreasing intracellular drug concentration, making antifungal therapy less effective. Biofilm development, which forms a protective matrix and alters cell physiology, may hinder antifungal drug penetration (Bultman et al., 2016). Mutations in genes involved in the ergosterol production pathway, as well as gene overexpression, can both contribute to antifungal resistance (Rajendran et al., 2013; Bultman et al., 2016). Other mechanisms include stress response pathways that assist the fungus in surviving antifungal therapy and heteroresistance, which occurs when a subset of cells is resistant to antifungal medications (Yang et al., 2021; Zubovskaia, 2025).

Concerns over *Aspergillus fumigates* are growing worldwide as resistant variants of the fungus become more common. In a 20-year surveillance study from China, triazole resistance in *A. fumigatus* fluctuated across periods: it decreased from 6.25% in 2005–2009 to 0.58% in 2010–2014, but subsequently increased to 8.43% in 2015–2019. Therefore, the apparent decline should be interpreted as a period-specific fluctuation rather than a sustained downward trend (Yang et al., 2021). The mutations TR34/L98H and TR46/Y121F/T289A are common resistance mechanisms that can be triggered by exposure to the environment. In addition to the inter-country spreading of triazole-resistant *A. fumigatus* on plant bulbs, patients can contract resistant strains of *A. fumigatus* by inhaling resistant conidia from the environment.

Several approaches are being investigated by researchers to counter *A*. *fumigatus* resistance. Lipopeptides and marine bioactive compounds have been found to be effective antifungal agents against triazole-resistant *A. fumigatus* isolates (Tashiro et al., 2025). To address resistance mechanisms, efflux pumps and CYP51A inhibitors are being developed (Tashiro et al., 2025). Combination therapy and immunotherapy can help reduce resistance development. Environmental and clinical surveillance can help identify high-resistance locations. Reduced fungicide use in agriculture and the development of alternative fungicides with different modes of action may help to slow the spread of resistance. Regular monitoring and surveillance of *A. fumigatus* isolates can aid in the identification of areas with high resistance prevalence. Furthermore, decreasing the use of azole fungicides in agriculture can decrease the emergence of resistance.

## 10 Biofilm biology, antifungal tolerance, and resistance intersections in *Aspergillus fumigatus*

Biofilm growth is increasingly recognized as a mechanistic basis for persistence across the chronic end of pulmonary aspergillosis and as a contributor to reduced antifungal responsiveness in airway- and cavity-associated disease. In this context, biofilms represent structured multicellular communities that adhere to biotic or abiotic surfaces and are embedded within a protective extracellular matrix, a growth mode that is broadly associated with enhanced tolerance to antimicrobial stress and immune effector pressure (Islam et al., 2024; Tashiro et al., 2025). In human lung disease, *A. fumigatus* biofilm is not diagnosed by a single routine clinical assay. Biofilm-associated disease is inferred from the combined presence of compatible structural lung disease, chronic cavitary or bronchiectatic radiology, persistent or repeated recovery of *Aspergillus* from sputum/BAL, positive *Aspergillus* IgG or galactomannan/PCR evidence where appropriate, and histopathologic demonstration of hyphal aggregates within necrotic or cavity material. In resected aspergilloma or cavitary lesions, GMS/PAS staining can show dense hyphal networks, while culture or PCR can support species-level attribution to *A. fumigatus*. Biomarkers such as galactomannan and molecular assays support detection of *Aspergillus* burden, but they do not by themselves prove biofilm architecture. Extracellular matrix components such as galactomannan, galactosaminogalactan, and extracellular DNA are biologically important in *A. fumigatus* biofilms, but most remain investigational rather than routine diagnostic markers for lung biofilm (Jabra-Rizk et al., 2004; Rather et al., 2021). This framework is clinically relevant because the same biological properties that stabilize fungal growth in environmental niches can stabilize persistence within bronchiectatic airways, cavitary lesions, and occasionally device-associated settings.

A defining feature of *A. fumigatus* biofilms is the extracellular matrix (ECM), which functions as both structural scaffold and protective interface. Mechanistically, the ECM can create a diffusion-limiting microenvironment and alter local physicochemical conditions, thereby reducing the effective exposure of hyphae to antifungal agents and host effector molecules, providing a biologically grounded explanation for treatment recalcitrance that extends beyond generalized concepts of “poor drug delivery” (Morelli et al., 2021). Experimental evidence indicates that extracellular DNA is a functional component of mature *A. fumigatus* biofilm ECM and contributes to antifungal resistance phenotypes, supporting a model in which ECM macromolecules directly shape drug response rather than acting as inert debris (Rajendran et al., 2013). Exopolysaccharides further link matrix biology to host interaction. Galactosaminogalactan has been shown to act as a dominant adhesin and to modulate host inflammatory recognition, indicating that ECM composition can influence both persistence and immune evasion (Rajendran et al., 2013).

Biofilm architecture also establishes physiologic heterogeneity that is difficult to capture with planktonic susceptibility testing. Nutrient and oxygen gradients partition the community into microdomains with distinct metabolic states. In *A. fumigatus*, hypoxic niches develop during biofilm maturation and have been experimentally linked to enhanced survival under antifungal pressure, supporting a tolerance model in which reduced killing can occur even without high-level resistance as defined by conventional MIC endpoints (Kowalski et al., 2020). This microenvironmental heterogeneity is consistent with clinical patterns of incomplete sterilization and relapse in chronic disease, particularly when therapy is prolonged and drug exposure is variable over time.

The clinical relevance of this growth mode is most apparent in chronic pulmonary aspergillosis and aspergilloma, where structurally abnormal lung niches provide the substrate for long-term fungal persistence. In cavities and bronchiectatic airways, matrix-embedded hyphal networks plausibly sustain chronic inflammation and reduce antifungal effectiveness, mechanistically bridging radiologic cavity disease with therapeutic difficulty (Morelli et al., 2021). A similar rationale applies to selected biomaterial-associated infections, where matrix-embedded growth on abiotic surfaces can amplify tolerance and complicate eradication, increasing the importance of source control when feasibleThese clinical links complement earlier descriptions that emphasize biofilm-associated protection from host immunity and antifungal drugs as a driver of chronic infection and inflammation (Donlan and Costerton, 2002).

Biofilm biology intersects directly with antifungal resistance mechanisms described elsewhere in the manuscript, but the key point in this section is that tolerance and resistance can coexist and may be mechanistically coupled. Biofilm-associated physiology can reduce drug penetration and alter fungal stress responses, while canonical resistance mechanisms further diminish effective drug exposure or target engagement. In *A. fumigatus*, resistance-associated processes described in biofilm settings include altered target availability, efflux activity, and matrix-related effects on drug access (Parker et al., 2014; Morelli et al., 2021). Environmental selection pressure from azole fungicide exposure remains an important upstream driver of resistant strain emergence, and this ecological dimension has been formally recognized in a multi-agency EFSA scientific assessment, which concluded that azole use outside human medicine can contribute to selection of azole-resistant *Aspergillus* species. The WHO has similarly highlighted the One Health linkage between antifungal use beyond human medicine and emerging resistance threats in *A. fumigatus*. At the mechanistic level, azoles inhibit lanosterol 14-α-demethylase (Cyp51A), and clinically important resistance is frequently associated with promoter tandem-repeat variants coupled with coding substitutions such as TR34/L98H and TR46/Y121F/T289A (Parker et al., 2014; Wiederhold et al., 2016). Efflux pumps can further reduce intracellular drug concentration, adding a complementary mechanism that can operate alongside target alterations (Holmes et al., 2016).

Because biofilm-associated persistence and resistance can both limit therapy, diagnostic approaches that identify disease early and provide actionable information about likely antifungal responsiveness are essential. In addition to imaging and conventional microbiology, biomarker and molecular assays can support early recognition and therapeutic targeting in high-risk settings. Galactomannan testing and other non-culture diagnostics are widely used as adjuncts in invasive disease, and they may also contribute to assessment in selected chronic settings when interpreted alongside clinical and radiologic context (Wang et al., 2019; Otu et al., 2023). Resistance-marker detection is increasingly relevant when azole resistance is suspected or when culture yield is low. A clinically validated example is the AsperGenius assay, which detects *Aspergillus* DNA and common azole-resistance markers directly from bronchoalveolar lavage samples, supporting earlier resistance awareness than culture-dependent workflows (Chong et al., 2015; 2016). This approach is particularly valuable in settings where phenotypic susceptibility testing is delayed or not feasible, and it complements the broader shift toward direct-from-sample molecular diagnostics discussed in later sections.

Therapeutic implications of biofilm-associated tolerance extend beyond conventional agent selection. Clinical management of biofilm-associated disease often requires prolonged therapy, and treatment is frequently constrained by toxicity, drug–drug interactions, and incomplete eradication in structurally abnormal niches, which can necessitate combined medical and surgical approaches in carefully selected patients (Holmes et al., 2016; Otu et al., 2023). Emerging antifungal candidates with novel mechanisms of action are being evaluated as potential options to address limitations of existing agents, including drugs targeting distinct metabolic pathways or delivery strategies that may improve lung exposure (Hites et al., 2016). Adjunctive strategies, including immunomodulation, combination regimens, and approaches aimed at disrupting biofilms, have also been explored as ways to reduce persistence and improve outcomes, although the evidence base remains evolving and syndrome-specific. Inhaled antifungal development illustrates a mechanistically aligned approach to airway disease, and PC945 has been investigated for activity against both azole-susceptible and resistant *A. fumigatus* strains, with findings that support further exploration of local delivery strategies in pulmonary infection contexts (Hites et al., 2016).

In summary, *A. fumigatus* biofilms provide a mechanistic explanation for chronicity and antifungal tolerance through ECM-mediated protection and physiologic heterogeneity, while resistance mechanisms and ecological selection pressures further reduce therapeutic reliability. Integrating biofilm biology with resistance-aware diagnostics and syndrome-appropriate therapy is therefore central to rational management and provides a biologically coherent bridge to the subsequent sections on molecular diagnostics, emerging therapeutics, and research roadmaps (Donlan and Costerton, 2002; Morelli et al., 2021).

## 11 Mechanism-aligned molecular diagnostics and therapeutic interpretation

### 11.1 Diagnostic logic across the pulmonary aspergillosis spectrum

A mechanism-aligned diagnostic strategy for *Aspergillus fumigatus* disease should not focus only on whether *Aspergillus* is detected, but on what the detection means in a specific host, lung environment, and clinicoradiologic syndrome. The same positive culture, PCR result, or biomarker signal may represent airway colonization, allergic bronchopulmonary aspergillosis, chronic pulmonary aspergillosis, or invasive pulmonary aspergillosis. Therefore, diagnostic interpretation must begin with the clinical phenotype and then integrate imaging, microbiology, histopathology, biomarkers, and molecular testing.

In allergic bronchopulmonary aspergillosis, diagnosis is primarily immunologic and clinicoradiologic. Fungal sensitization, total IgE, *A. fumigatus*-specific IgE or IgG, eosinophilia, mucus plugging, and bronchiectatic changes are more informative than culture alone, because the key pathogenic process is allergic airway inflammation rather than tissue invasion (Agarwal et al., 2024). In chronic pulmonary aspergillosis and aspergilloma, chronicity, compatible thoracic imaging, pre-existing structural lung disease, and supportive mycologic or serologic evidence are central. In invasive pulmonary aspergillosis, particularly in neutropenic, transplant, or intensively immunosuppressed patients, diagnosis is time-sensitive and should combine host risk, compatible imaging, and mycologic evidence according to established consensus frameworks (Patterson et al., 2016; Peter Donnelly et al., 2020).

This syndrome-based logic is important because it prevents overinterpretation of isolated test results. Respiratory culture or PCR positivity may reflect colonization in structurally abnormal airways, whereas the same finding in a high-risk immunocompromised host with compatible CT abnormalities may support urgent treatment for invasive disease. Thus, molecular diagnostics are most useful when they are interpreted as part of an integrated diagnostic pathway rather than as stand-alone tests.

### 11.2 Complementary roles of culture, histopathology, biomarkers, PCR, and sequencing

Conventional microbiology and tissue-based pathology remain essential even as molecular platforms expand. Culture from sputum, bronchoalveolar lavage, or tissue can support species identification and enables antifungal susceptibility testing, which is especially important in the setting of rising azole resistance. However, culture sensitivity varies by specimen quality, fungal burden, and prior antifungal exposure, and a positive respiratory culture does not by itself prove invasive disease. Histopathology, particularly with Gomori methenamine silver or periodic acid–Schiff staining, can demonstrate tissue invasion, vascular invasion, necrosis, and host response pattern, but hyaline septate mold morphology is not fully species-specific and should be correlated with culture or molecular identification when possible (Guarner and Brandt, 2011; Barnes et al., 2018).

Non-culture biomarkers provide earlier diagnostic support in appropriate populations. Galactomannan is more closely associated with *Aspergillus* burden, whereas β-D-glucan is a broader invasive fungal disease marker and is not specific for *Aspergillus*. Serum and bronchoalveolar lavage galactomannan can support the diagnosis of invasive aspergillosis, particularly when interpreted with host risk and imaging findings. β-D-glucan is best used as an adjunctive marker rather than as a species-specific diagnostic tool (Lehrnbecher et al., 2016; Patterson et al., 2016).

PCR-based assays add organism-level detection and may shorten time to diagnosis, particularly when culture is negative or delayed. Assays targeting *Aspergillus* DNA in bronchoalveolar lavage, blood, or tissue can increase diagnostic confidence when used with biomarkers and imaging. Molecular sequencing of conserved fungal loci, including ITS or ribosomal targets, can also support species-level identification from tissue when culture is unavailable or compromised. More recently, metagenomic next-generation sequencing and breath volatile organic compound profiling have been explored as emerging diagnostic platforms, but these approaches require further validation, standardization, and disease-specific performance evaluation before they can replace established diagnostic pathways (Koo et al., 2014; Barnes et al., 2018; Xu et al., 2025).

### 11.3 Biofilm-aware diagnostic interpretation in human lung disease

Biofilm-associated *A. fumigatus* disease should be interpreted carefully because there is no single routine clinical assay that directly diagnoses pulmonary *A. fumigatus* biofilm. In human lungs, biofilm-associated disease is usually inferred from a combination of structural lung disease, chronic cavitary or bronchiectatic radiology, persistent or repeated recovery of *Aspergillus* from respiratory specimens, compatible serologic or molecular evidence, and histopathologic demonstration of dense hyphal aggregates in necrotic or cavity material.

In resected aspergilloma, cavitary lesions, or biopsy material, GMS and PAS staining can show organized hyphal networks within mucus, necrotic debris, or cavity contents. Culture or PCR from the same material can support species-level attribution to *A. fumigatus*. However, galactomannan, PCR, and culture primarily demonstrate the presence or burden of *Aspergillus*; they do not by themselves prove biofilm architecture. Extracellular matrix components such as galactomannan, galactosaminogalactan, and extracellular DNA are biologically important in *A. fumigatus* biofilm development, but most remain research tools rather than routine clinical markers of lung biofilm. Therefore, biofilm-associated pulmonary aspergillosis should be described as a clinicopathologic and microbiologic inference rather than as a diagnosis made by one test alone.

This distinction has therapeutic relevance. Biofilm-associated growth can reduce antifungal exposure, promote physiologic heterogeneity, and contribute to persistence in cavities and bronchiectatic airways. Diagnostic recognition of this pattern supports prolonged therapy, resistance testing where feasible, and consideration of surgical or source-control approaches in selected patients with localized aspergilloma, refractory cavitary disease, or life-threatening hemoptysis.

### 11.4 Resistance-marker detection and direct-from-sample testing

Resistance-aware diagnostics are increasingly important because azole-resistant *A. fumigatus* can emerge during prolonged patient therapy or be acquired from environmental exposure. The most widely recognized resistance mechanisms involve *cyp51A* alterations, including tandem-repeat promoter variants coupled with coding substitutions such as TR34/L98H and TR46/Y121F/T289A. However, resistance may also involve non-*cyp51A* mechanisms, efflux activity, stress-response pathways, or other genetic changes, so a negative result for common markers does not exclude clinically relevant resistance (Parker et al., 2014; Wiederhold et al., 2016; Fisher et al., 2022).

Direct-from-sample assays that detect *Aspergillus* DNA and common azole-resistance markers can provide earlier resistance awareness than culture-dependent workflows. The AsperGenius assay, for example, has been evaluated for detection of *Aspergillus* DNA and resistance-associated mutations directly from bronchoalveolar lavage fluid (Chong et al., 2015). These tools are especially useful when culture yield is low, when prior antifungal exposure reduces recovery, or when rapid therapeutic adjustment is needed. Nevertheless, molecular resistance tests should complement rather than replace culture-based susceptibility testing, because molecular panels usually target selected known mechanisms and may miss uncommon or emerging resistance pathways.

### 11.5 Therapeutic implications of diagnostic results

The clinical value of molecular diagnostics lies in their ability to support earlier and more appropriate therapy. In suspected invasive pulmonary aspergillosis, compatible imaging and mycologic evidence should prompt early mold-active therapy while additional confirmation is pursued, because delays in treatment are associated with poor outcomes. Voriconazole remains a central first-line option for invasive aspergillosis, with isavuconazole or liposomal amphotericin B used in selected settings depending on toxicity, drug interactions, susceptibility, and local resistance patterns (Patterson et al., 2016).

In chronic pulmonary aspergillosis, diagnostic evidence should guide long-term oral azole therapy, monitoring for toxicity, drug–drug interactions, therapeutic drug levels, and emergence of resistance. In allergic bronchopulmonary aspergillosis, immunologic markers and imaging guide anti-inflammatory therapy and antifungal adjuncts rather than urgent invasive-disease management. Thus, diagnostic tests should be interpreted with the clinical syndrome and linked to treatment decisions, as summarized in the diagnostic table. A positive test should answer not only “Is *Aspergillus* present?” but also “Which disease phenotype is present, is tissue invasion likely, is resistance possible, and what therapy is justified?”

## 12 Translational gaps and experimental roadmap

A major limitation in *A. fumigatus* research is that experimental models often emphasize acute invasive disease under profound immunosuppression, whereas human aspergillosis spans allergic airway disease, chronic cavity-associated infection, subacute invasive disease, and rapidly progressive angioinvasive infection. A single model cannot represent all of these syndromes. Future studies should therefore match the experimental system to the disease phenotype being investigated. Allergic bronchopulmonary aspergillosis requires models that reproduce fungal sensitization, Th2-skewed inflammation, eosinophilia, and mucus pathology. Invasive aspergillosis studies require models of neutropenia, corticosteroid exposure, transplant-like immunosuppression, or other defined host defects. Chronic pulmonary aspergillosis and aspergilloma require models that better approximate structural lung disease, cavitary niches, chronic inflammation, and biofilm-associated persistence (Kurup and Grunig, 2002; Desoubeaux and Cray, 2018).

Model selection should also be tied to meaningful endpoints. For invasive disease, survival, fungal burden, angioinvasion, tissue necrosis, and antifungal response remain important. For chronic disease, relevant endpoints include persistence, cavity-associated growth, biofilm matrix formation, relapse after treatment interruption, and drug tolerance. For host–pathogen interaction studies, transparent systems such as larval zebrafish can help visualize phagocyte recruitment, fungal clearance, and early invasion dynamics in real time, while mammalian models remain necessary for evaluating lung architecture, pharmacokinetics, and clinically relevant immune defects (Knox et al., 2017).

A second translational gap is resistance surveillance. Culture-dependent surveillance can underestimate resistance when respiratory cultures are negative, when antifungal exposure suppresses growth, or when resistant subpopulations are missed. Direct molecular detection of resistance markers from bronchoalveolar lavage or tissue may improve early recognition, but broader surveillance must include both clinical and environmental isolates. A One Health framework is needed because environmental azole exposure can select resistant *A. fumigatus* strains that may subsequently infect patients through inhalation. Future surveillance should therefore integrate clinical microbiology, agricultural azole-use data, environmental sampling, and standardized resistance-marker reporting.

A third priority is identification of mechanistic nodes that connect virulence, persistence, and drug tolerance. Biofilm extracellular matrix biology is one such node because matrix components can promote adhesion, immune evasion, antifungal tolerance, and chronicity. Stress-response pathways, including MAPK-linked adaptation, hypoxia responses, iron acquisition, and cell-wall integrity signaling, are also attractive because they connect host-imposed stress with survival and treatment response. Similarly, resistance mechanisms beyond canonical *cyp51A* mutations, including efflux regulation and non-*cyp51A* genetic pathways, should be incorporated into diagnostic and drug-discovery strategies rather than treated as secondary exceptions.

Finally, diagnostic innovation should be tested in clinically realistic pathways. New assays should be evaluated separately for ABPA, chronic pulmonary aspergillosis, and invasive pulmonary aspergillosis rather than pooled across biologically distinct syndromes. Future studies should report how each diagnostic tool changes clinical decisions, time to therapy, antifungal selection, need for biopsy, detection of resistance, and patient outcome. This implementation-focused approach is necessary to move from technologically advanced testing to clinically useful testing.

## 13 Emerging therapeutic directions and translational priorities

Future therapy for *A. fumigatus* disease should be framed around three linked challenges: increasing antifungal resistance, limited activity of existing drugs in biofilm-associated or structurally abnormal lung niches, and host-specific vulnerability caused by immune suppression or chronic lung disease. Emerging treatment strategies should therefore not be presented simply as a list of new drugs. Instead, they should be evaluated according to mechanism of action, expected clinical niche, resistance profile, toxicity, pulmonary exposure, and evidence stage.

Novel antifungal classes are especially important because the current treatment landscape remains dependent on triazoles, amphotericin B formulations, and echinocandins. Olorofim, an orotomide antifungal that targets fungal dihydroorotate dehydrogenase, represents a non-azole approach with potential relevance for azole-resistant *Aspergillus* infection. Fosmanogepix, a prodrug of manogepix, targets Gwt1-dependent glycosylphosphatidylinositol-anchor biosynthesis and has been evaluated for invasive mold diseases caused by *Aspergillus* species and rare molds. Glucan-synthase-targeting agents, including rezafungin and ibrexafungerp, illustrate continued interest in cell-wall-directed therapy, although their role in aspergillosis should be described cautiously and distinguished from established first-line therapy. Modified polyenes and amphotericin B formulations are also being explored to preserve broad mold activity while reducing toxicity or improving delivery (Jallow and Govender, 2021; Vanbiervliet et al., 2024).

Pulmonary drug delivery is another rational direction because the lung is the dominant site of *A. fumigatus* disease. Inhaled antifungal approaches aim to achieve high local airway or parenchymal exposure while limiting systemic toxicity and drug–drug interactions. This strategy is conceptually attractive for prophylaxis, chronic airway disease, and selected invasive pulmonary disease contexts. However, inhaled agents such as PC945/opelconazole should be described as investigational rather than as established therapy, and their clinical role should be interpreted cautiously until robust outcome data define patient selection, safety, and efficacy.

Combination therapy remains an important but unresolved area. Combining mold-active azoles, amphotericin B formulations, or echinocandins may be considered in selected severe, refractory, or resistant cases, but routine combination therapy is not established for all patients with invasive aspergillosis. Future combination studies should be mechanism-driven rather than empirical. For example, combinations could be selected to improve activity against resistant isolates, enhance cell-wall stress, improve biofilm penetration, or pair antifungal therapy with host-directed immune support. These studies should use clinically relevant endpoints, including survival, fungal burden, resistance emergence, relapse, and toxicity.

Biofilm-directed therapy is another translational priority. Because extracellular matrix, extracellular DNA, galactosaminogalactan, hypoxia, and altered metabolic states contribute to antifungal tolerance, future strategies may include matrix-disrupting agents, improved local drug delivery, or adjunctive therapies that increase antifungal penetration and immune clearance. At present, these approaches remain largely investigational, and they should be presented as research priorities rather than routine clinical options. Their greatest potential relevance is likely to be chronic pulmonary aspergillosis, aspergilloma, bronchiectatic airway colonization with repeated exacerbations, and device-associated infection.

Host-directed therapy and vaccination also remain important research directions. Immunomodulatory strategies such as G-CSF, GM-CSF, interferon-γ, monoclonal antibodies, or adoptive cellular approaches may be relevant in carefully selected immunocompromised patients, but they require syndrome-specific evaluation because excessive inflammation may worsen tissue injury in allergic or chronic inflammatory phenotypes. Vaccine development is biologically plausible, particularly for high-risk groups, and experimental studies using live or heat-killed mutant conidia have suggested protective potential in preclinical models. However, no vaccine is currently part of routine clinical management, and future work must define protective antigens, immune correlates, safety in immunocompromised hosts, and target populations (Fernandes et al., 2022).

The long-term goal is personalized management of aspergillosis. Such an approach would integrate host risk, lung architecture, disease phenotype, fungal species identification, resistance markers, biofilm-associated persistence, drug exposure, toxicity risk, and immune status. In this framework, diagnostics and therapy are not separate domains: rapid molecular diagnosis supports early treatment, resistance detection guides antifungal selection, biomarkers help monitor response, and mechanistic understanding identifies when prolonged therapy, surgery, immune support, or investigational strategies may be justified. This integrated model provides a more clinically useful direction for future research than a non-specific list of emerging technologies.

## 14 Conclusion

Recent research has clarified the molecular basis of pulmonary aspergillosis by linking airway epithelial sensing, innate immune failure, fungal virulence programs, biofilm-associated persistence, and antifungal resistance. *Aspergillus fumigatus* pathogenesis depends on dynamic interactions between inhaled conidia, host immune status, lung architecture, and fungal stress-adaptation pathways. The increasing recognition of azole-resistant *A. fumigatus*, including environmentally selected resistant strains, highlights the need for integrated clinical and environmental surveillance. Emerging agents such as olorofim, fosmanogepix, rezafungin, ibrexafungerp, and newer amphotericin B formulations remain investigational or context-dependent, and their future role should be defined through syndrome-specific clinical evidence.

According to research, epithelial cells play a critical role in identifying and reacting to A. fumigatus, initiating immunological reactions that either eradicate the fungus or cause tissue harm. This review emphasizes the range of *A. fumigatus* infections, spanning from allergic responses to possibly deadly invasive aspergillosis. The challenges in diagnosing *A. fumigatus* infections due to the limitations of current diagnostic tools emphasize the demand for improved biomarkers and molecular diagnostics. The increasing occurrence of antifungal resistance in *A. fumigatus* necessitates the development of new treatment methods. Grasping *A. fumigatus* infections will enable us to create improved preventive, diagnostic, and treatment approaches, ultimately aiding individuals afflicted by the related severe diseases.
